# Optimal Design of Plant Canopy Based on Light Interception: A Case Study With Loquat

**DOI:** 10.3389/fpls.2019.00364

**Published:** 2019-03-26

**Authors:** Liyu Tang, Dan Yin, Chongcheng Chen, Dayu Yu, Wei Han

**Affiliations:** ^1^Key Laboratory of Spatial Data Mining and Information Sharing of Ministry of Education, Fuzhou University, Fuzhou, China; ^2^National Engineering Research Center of Geospatial Information Technology, Fuzhou University, Fuzhou, China

**Keywords:** canopy architecture, interactive shaping and pruning, light distribution, light interception, silhouette to total area ratio (STAR), net photosynthetic rate

## Abstract

Canopy architecture determines the light distribution and light interception in the canopy. Reasonable shaping and pruning can optimize tree structure; maximize the utilization of land, space and light energy; and lay the foundation for achieving early fruiting, high yield, health and longevity. Due to the complexity of loquat canopy architecture and the multi-year period of tree growth, the variables needed for experiments in canopy type training are hardly accessible through field measurements. In this paper, we concentrated on exploring the relationship between branching angle and light interception using a three-dimensional (3D) canopy model in loquat (*Eriobotrya japonica Lindl*). First, detailed 3D models of loquat trees were built by integrating branch and organ models. Second, the morphological models of different loquat trees were constructed by interactive editing. Third, the 3D individual-tree modeling software LSTree integrated with the OpenGL shadow technique, a radiosity model and a modified rectangular hyperbola model was used to calculate the silhouette to total area ratio, the distribution of photosynthetically active radiation within canopies and the net photosynthetic rate, respectively. Finally, the influence of loquat tree organ organization on the light interception of the trees was analyzed with different parameters. If the single branch angle between the level 2 scaffold branch and trunk is approximately 15° and the angles among the level 2 scaffold branches range from 60 to 90°, then a better light distribution can be obtained. The results showed that the branching angle has a significant impact on light interception, which is useful for grower manipulation of trees, e.g., shoot bending (scaffold branch angle). Based on this conclusion, a reasonable tree structure was selected for intercepting light. This quantitative simulation and analytical method provides a new digital and visual method that can aid in the design of tree architecture.

## Introduction

Canopy architecture has a strong impact on light interception, water transport and transpiration, as well as carbon acquisition and allocation (Silva et al., 2014b; Burgess et al., 2017; Gao et al., 2018). Therefore, many scholars have sought to determine optimal plant architecture, i.e., the ideotype (Rpa et al., 2018). In the field of fruit tree training, knowledge of the geometrical and topological characteristics of fruit trees is helpful for better adjusting the shape of fruit trees, thereby improving fruit production in terms of quantity, regularity and quality (Rosell and Sanz, 2012). Therefore, the optimization of tree architecture has always been considered the ideal choice for fruit tree culture. Loquats (*Eriobotrya japonica Lindl*), which are characterized by a soft pericarp, juiciness, a pleasant taste, nutritional richness, and a high pharmacological value, are favored in markets worldwide (Zhou et al., 2018). As a kind of evergreen fruit tree, loquat trees exhibit very strong vigor. Naturally growing loquat trees are often tall. If the crown is not pruned, it will be too tall, its inner chamber will be too dense, the fruit will be produced near the outside, the flowers and fruits will be easily frozen and sunburned, and the yield will be low. It is necessary to dwarf loquat trees by shaping and pruning them, thereby improving ventilation and light transmission, reducing the occurrence of diseases and insect pests, and cultivating strong fruiting branches to increase the yield (Yuan et al., 2014). Traditional shaping and pruning used to adjust the crown are mainly based on empirical knowledge, which has revealed that these changes are irreversible and affected by the environment, manpower, physics, and other factors. With the development of information technology, virtual plant technology can provide information services and technical support for digital modeling, growth process simulation (Tang et al., 2015a), analysis of light interception efficiency and so on within the agricultural and forestry industries. In this paper, the relationship between loquat architectural parameters and the distribution of light is simulated and analyzed with a three-dimensional (3D) model to explore the optimum distribution of light in the trees.

In the last 20 years, various virtual plant geometric modeling approaches have been proposed, and the corresponding technology and software have been developed (Prusinkiewicz and Lindenmayer, 1990; Quan et al., 2006; Sonohat et al., 2006), e.g., geometry-based, image-based, and rule-based modeling and 3D construction based on digitally measured data. According to model feature requirements, virtual plant models can be divided into three categories: pure graphical models focusing on visual effects; static structural models with an emphasis on morphological characteristics; and dynamic structural models with an emphasis on growth laws. These models are constructed with various levels of precision. The models with low precision are simple but not suitable for analysis. Instead, high-precision models are more suitable than low-precision ones for such analysis. Virtual 3D tree pruning offers users realistic means to design various canopy types. The tree modeling system should have functions for manipulating components of the model and editing the model. In the present study, a parameterized L system and organ modeling based on point cloud data were used to construct a detailed canopy model. Furthermore, we focused on a 3D model with interactive editing.

Light regulates photosynthesis, growth, morphogenesis, substance metabolism, and gene expression in plants (Lin et al., 2017). Therefore, light interception is considered a major performance metric for defining ideotypes (Silva et al., 2014b; Gao et al., 2018; Rpa et al., 2018). An optimal canopy architecture is generally evaluated using parameters such as leaf shape (Rpa et al., 2018), leaf angle, leaf direction (Jung et al., 2018; Ventrelespiaucq et al., 2018), internode length (Silva et al., 2014b) and so on. Scaffold branch angle is one of the most widely used parameters in fruit training systems, because the adjustment of the angle of the branches will change the direction of the leaf correspondingly. However, it is difficult to take detailed measurements in the field to evaluate light interception within a canopy. Virtual plants are a novel tool for analyzing light distribution and light interception. Virtual plants can be used to quantitatively describe plant topology, geometry, and organ position. Plant organs can be represented in small 3D units, and radiation transmission and interception among the units can be simulated with ray tracing (Génard et al., 2000; Tang et al., 2015b; Hoon et al., 2016) or a radiosity algorithm (Chelle and Andrieu, 1998; Wiechers et al., 2011; Tang et al., 2017). In recent years, the exploration of plant light distribution with computers has mainly involved crops and has made substantial, valuable progress (Liu et al., 2011; de Visser et al., 2014; Wang et al., 2017); however, there are few reports on fruit trees. In fruit trees, the total leaf area (TLA) and silhouette to total area ratio (STAR) are considered key performance metrics representing light interception (Silva et al., 2014a,b; Pallas et al., 2016; Yang et al., 2016). Additionally, the net photosynthetic rate (P_n_) has been considered a key variable in light interception.

The objective of this study is to investigate the effects of different geometric and topological structures on light distribution to reveal a reasonable tree structure with high light interception in loquat. The main steps are as follows: (1) construct a detailed 3D loquat structural model, (2) implement interactive shaping and pruning, which can be used to rapidly construct different geometric forms of loquat, and (3) simulate the light distribution and analyze the light interception within a loquat canopy at different scales.

## Materials and Methods

Based on previous studies using the individual-tree modeling software LSTree (Lin Y. et al., 2011; Tang et al., 2011), it is possible to build a precise 3D canopy model according to the topology and geometry of the loquat tree canopy. By interactively pruning branches and adjusting angles, various loquat tree models were generated. A radiosity algorithm and modified rectangular hyperbola model were used to simulate the distribution of photosynthetically active radiation (PAR) and estimate the P_n_, respectively. The STAR, P_n_, etc. were used to analyze light interception.

### Measurement of Morphological Parameters

The loquat architectural parameters were measured in Yunxiao County, Fujian Province, China, on January 18-22, 2016. We randomly selected a flat form of “Zaozhong No. 6” loquats for investigation. Based on investigation and measurement in the field or reference to the literature (Zheng et al., 2006), we defined the morphological structural parameters of the loquat trees (Table 1). We measured and statistically analyzed the tree height, crown diameter, central stem length and diameter, central shoot length and thickness, lateral shoot length and thickness, and number of leaf blades by means of a tape measure, ruler and protractor. At the same time, we captured point cloud data of the loquat canopy organs by a hand-held structured light scanner (Artec Eva, America).

**Table 1:** Main morphological and structural parameters of loquat trees.

Height of tree (m)	East and west of crown (m)	South and north of crown (m)	Thickness of leaf canopy (m)	Trunk cross-sectional area (m^2^)
1.35	2.31	2.364	1.30	0.025

### 3D Construction of Models

**Figure 1:**
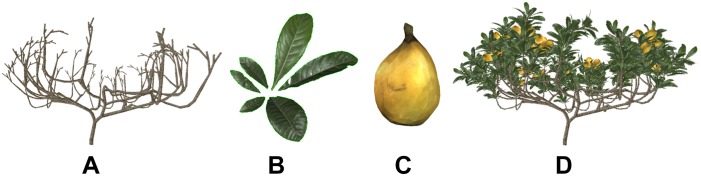
Simulated a loquat tree and the model representation for each part. **(A)** Branch model built based on the L-system; **(B,C)** leaf model and fruit model built based on point cloud data; **(D)** a detailed 3D model integrating the loquat organ models.

We employed parametric L-system rules to construct 3D tree models. The two core steps included defining the structure of the basic unit according to the self-similarity of shape and specifying L-system rules for the branch system consisting of axiom and production rules. Since the same species of trees have similar branch shapes and structures, the 3D branch models are described to establish a branch library, and the corresponding simple alphanumeric symbols are expressed. In this way, the efficiency of the 3D reconstruction of tree architecture can be improved. A set of shape types of loquat branches was summarized, and the corresponding 3D tree branch patch library was established. Figure 1A depicts the main branch system model, consisting of subgraphs. If the branch patches in the library do not match the real-world tree branch shape, then new branches should be generated by adjusting and modifying the rule of existing branches in the library and the branch parameters derived from measurements.

Tree canopy organs include branches, leaves, fruits, etc. To build a canopy organ model with high precision, the canopy was reconstructed based on point cloud data. Preprocessing point cloud data with arctec studio9.1 software for data fusion and noise removal. The Delaunay triangulation algorithm was employed to generate 3D mesh models of the organ, and for mapping the texture, a realistic and precise organ model was built. The leaf model and fruit model are shown in Figure 1B,C. Based on the geometric relationship between the canopy shoot and the trunk, we defined the growth knots of the shoots on branches and then attached shoots to the main branch system. Figure 1D shows a detailed 3D loquat tree model. In the subsequent simulation of light distribution, we focused on leaves influencing light interception; therefore, the fruit model was not integrated into the tree model in the processes of light distribution simulation.

### Interactive Shaping and Pruning

Interactive shaping and pruning of virtual plants provide intuitionistic and flexible methods for dynamic adjustment and modification of geometric models. This process provides a method of digital and visual expression for plant type design in agricultural and forestry management, e.g., plant growth regulation. Therefore, interactive shaping and pruning simulation is an important topic of research in digital forestry and digital agriculture. However, interactive shaping and pruning methods for 3D tree models are rarely reported in the literature. Natural trees usually have 1000s of organs, and a detailed 3D tree model therefore consists of a large number of organ units. Interactive shaping and pruning manipulation of virtual plants should meet the following conditions: (1) the branches to be manipulated can be chosen accurately and quickly; (2) the manipulation conforms to the topological relationships within the tree branching system; and (3) the interface is user friendly, and the human-machine interaction process mimics real manual pruning.

**Figure 2:**
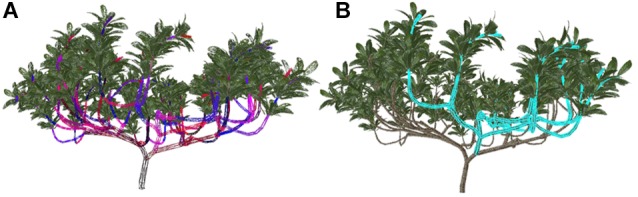
The key steps in the process of selecting branches and schematic diagram after selection. **(A)** According to the IDs of branch models drawn with false colors, the model is rendered offscreen. **(B)** The highlighted parts indicate the selected branch and sub-branches.

To select the shaping and pruning objects quickly and accurately from a large number of branches in a complex tree model, we used a frame buffer object (FBO) and pseudocoloring to render the whole tree model offscreen (Lin D. et al., 2011) (Figure 2A). Then, the pixel values under the mouse were sampled to achieve the rapid selection of a very large number of image elements (Figure 2B). Finally, the process of shaping and pruning took place when the parameters in the interactive interface were adjusted according to the selected objects and the adjusted tree shapes were drawn. To facilitate data organization, management and operation, the rules of the model were stored in a linked list, and the branch data of the model were stored separately in a container according to level, e.g., the trunk was level 0, which produced the branches in level 1. The identity of the branch unit was denoted by a serial number. The character ‘F’ in the rule is the basic unit of the stem model. The structure represented by the character ‘F’ is taken as the basic operation object in the acquisition and feedback of the structure parameter information.

The implementation of shaping and pruning is as follows:

First, trees are divided into the editable state and the non-editable state. In the non-editable state, no operations can be performed on the tree, and the tree is displayed normally. In the editable state, the 3D model of the tree is rendered on the screen with normal colors and textures, and the model is visible; at the same time, in color index mode, the tree model is repainted according to different branch segments using pseudocolor rendering to the offscreen buffer of the graphics processing unit (GPU) using a 32-bit alignment technique. The invisible tree model is used for selection.

Second, when the object unit is selected, the type is judged according to the coordinates under the mouse click. If the selected type is 0, then the tree model in the scene is not selected; if the selected type is 1, then a branch is selected. The color-coding values of the selected stem units are decoded to obtain the branch ID. By using hierarchical relationships, all sub-branches of the branch can be obtained and marked as selected. At the same time, OpenGL rendering technology and recursive invocation are used to highlight the selected units that are to be edited.

Finally, the linked list of character parsing results is traversed after the completion of selection. According to the selected branch ID, the position is found, which corresponds to the rule character ‘F.’ Then, the parameters of the node in the parsed list are adjusted, and the result list is updated. The tree model is rendered on the screen after editing, such as pruning and changing the angle and length of branches. Through editing operations, according to the topological structure of the tree model branches, the information on branches, leaves, flowers, fruits, and other organs attached to the branches is adjusted or deleted. These processes can simulate plastic manipulation such as pulling branches in real tree management and pruning in maintenance management.

### PAR Distribution Simulation

Radiative transfers are essential conditions in the process of plant growth and development (Chelle and Andrieu, 1998). However, adequately measuring light is time consuming due to heterogeneity in the arrangement and composition of the canopy, diurnal and seasonal changes in solar position and cloudiness, and leaf on and off cycles (Stadt and Lieffers, 2000). Therefore, it is very important to use a suitable and effective method to analyze the light distribution in the canopy. At present, the ray tracing algorithm (Ross and Marshak, 1988) and radiosity algorithm (Sparrow, 1963; Goral et al., 1984) are alternative methods that are useful for a wide variety of radiation transport problems. Goel and Strebel (1984) and Borel et al. (1991) proposed radiosity methods adapted to canopies. The radiosity equation is as follows:

$$B_{i}\;=\;E_{i}\;+\;{\text{ρ}}_{i}\;\sum_{j=1}^{N}\;B_{i}F_{ji}(i\;=\;1,2,\cdots ,N)\;\;\;\;\;\;\;\;\;\;\;\;\;\;(1)$$

where *B_i_* is the radiosity of facet *i* in a scene, that is, the total light energy exiting facet *i* per unit area per unit time; *E_i_* is the emittance of facet *i*; ρ_*i*_ is the diffuse reflectivity; *N* is the number of surfaces in the scene; and *F_ji_* is the form factor between surface *i* and surface *j*, which describes the contribution of each facet *j* to the radiosity at facet *i*.

The incoming radiation is partitioned into solar direct radiation and sky diffuse radiation. The direct light source is described as a reference projection plane located above the canopy, normalized to the direction of light, and is discretized into triangles. The light source from the sky is described as the sky hemisphere over the canopy and is divided by latitude and longitude. The construction of a concrete light source may be achieved by referencing our previous work (Tang et al., 2017). According to the energy provided by the light source, the 3D discrete view factor (3D-DVF) method is used to solve the radiosity equation. The calculation of the form factor is costly in terms of computation. Paralleling this algorithm is a good way to significantly reduce computation time (Renaud and Rousselle, 1997). Therefore, the radiosity algorithm based on CUDA (Hou et al., 2015; Tang et al., 2018) was used to simulate direct PAR and diffuse PAR within tree models at various scales in the present research.

### Light Interception Characteristics

To evaluate light interception efficiency, we used the STAR, PAR, and P_n_. The STAR, where silhouette area refers to the projected leaf area on a plane that is perpendicular to the projective direction (Silva et al., 2014a). P_n_ that is net photosynthetic rate. The STAR is the relative irradiance of the leaf area, which can be calculated by

$$STAR\;=\;\frac{PLA}{TLA}\;\;\;\;\;\;\;\;\;\;\;\;\;\;\;\;\;(2)$$

where *PLA* is the projected leaf area, i.e., the silhouette area of the tree, and *TLA* is the total leaf area, i.e., 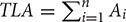, where A_i_ is the area of leaf *i* and *n* is the total number of leaves. In the present research, the tree model is projected onto the “ground” by matrix transformation, and the shadow is then shown. The value of the *PLA* is also the area of the shadow of the blade. This value is easy to calculate with the organ coordinates and branch coordinates, which are stored separately when modeling. *TLA* is the sum of all the triangular areas of the leaf.

The response of photosynthesis to light is of fundamental importance for understanding the photochemical efficiency of the process (Sharp et al., 1984). To date, many light response models of photosynthesis have been established by many scholars (Farquhar et al., 1980; Wullschleger, 1993; De et al., 1997) and have been applied to fitting the light response curves of various plants. At present, the non-rectangular hyperbola model is widely used to study the photosynthetic characteristics of plants (Cannell and Thornley, 1998; Liu et al., 2005). However, the model cannot estimate the saturated light intensity of plants, and the estimated maximum net photosynthetic rate deviates greatly from the measured value; therefore, the modified rectangular hyperbola model (Ye, 2007; Ye and Yu, 2007) of the response of photosynthesis to light was developed. The fitting results show that the model is more practical than other light response models and is consistent with the measured values (Wu et al., 2011; Yan et al., 2013). In the present study, the modified rectangular hyperbola model was used to simulate the P_n_ of loquat. The mathematical model is shown in formula (3).

$$P_{n}(I)\;=\;\text{α}\frac{1\;-\;\text{β}I}{1\;+\;\gamma I}I\;-\;R_{d}\;\;\;\;\;\;\;\;\;\;\;\;\;\;\;\;\;\;\;(3)$$

where *P_n_* is the net photosynthetic rate; α is the initial slope of the light response curve, i.e., the apparent quantum efficiency (*AQE*); *β* is the correction coefficient; γ is the ratio of the α to the maximum P_n_ (P_italicmax_) of the plants, i.e., γ = α/P_italicmax_; R_d_ is the dark respiration rate; and *I* is the PAR. The values of the α, β, γ, *R_d_*, P_italicmax_, light saturation point (*LSP*), light compensation point (*LCP*) and so on can be obtained by equation fitting. The formulas for calculating the related parameters are as follows:

$$P_{\max}\;=\;\text{α}{\left(\frac{\sqrt{\text{β}+}\gamma \;-\;\sqrt{\text{β}}}{\gamma }\right)}^{2}\;-\;R_{d}\;\;\;\;\;\;\;\;\;\;\;\;\;\;\;\;\;\;\;\;\;\;\;\;\;\;\;\;\;\;\;\;(4)$$

$$LSP\;=\;\frac{\sqrt{\text{β}+}\gamma /\sqrt{\text{β}}\;-\;1}{\gamma }\;\;\;\;\;\;\;\;\;\;\;\;\;\;\;\;\;\;\;\;\;\;\;\;\;\;\;\;\;\;\;\;\;\;\;\;\;\;\;\;\;\;\;\;\;\;\;\;\;\;\;\;\;\;\;(5)$$

$$LCP\;=\;\frac{\text{α}\;-\;R_{d}\;-\;\sqrt{{\left(\text{α}\;-\;R_{d}\right)}^{2}\;-\;4}\alpha \beta R_{d}}{2\alpha \beta }\;\;\;\;\;\;\;\;\;\;\;\;\;\;\;\;\;\;\;\;(6)$$

According to the literature (Yang and Li, 2016), the parameters of the light response curve of loquat trees are shown in Table 2. The values of parameters α, β, and γ are calculated from formulas (4), (5), and (6) are: 0.065, 2.02 × 10^-4^, 0.0074. Then, the PAR value of each leaf is obtained accurately using virtual canopy solar direct radiation and sky diffuse radiation simulation techniques. The P_n_ of the loquat canopy at different times was obtained by formula (3) using the corresponding PAR values.

**Table 2:** Light response curve correlation parameter.

P_italicmax_ (μmol ⋅ m^-2^ ⋅ s^-1^)	*R_ d_* (μmol ⋅ m^-2^ ⋅ s^-1^)	*LCP* (μmol ⋅ m^-2^ ⋅ s^-1^)	*LSP* (μmol ⋅ m^-2^ ⋅ s^-1^)	*AQE* (μmol ⋅ m^-2^ ⋅ s^-1^)
8.794	0.327	4.535	693.799	0.065

## Results

### At the Branch Scale

#### Branch and Trunk

**Figure 3:**
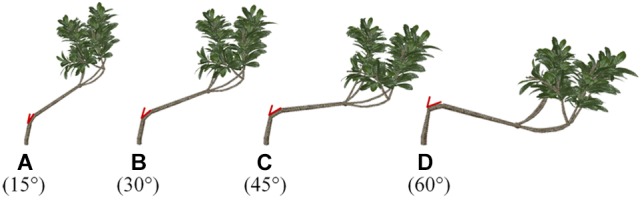
**(A–D)** Schematic representation of different branches with different angles with respect to the trunk. The red part of the picture shows the angle in three-dimensional space, that is, the angle between the main direction and the level 2 scaffold branch.

One branch of the loquat models was chosen arbitrarily as the adjustable object for shaping and pruning. Then, the angle between the branch and the trunk was changed (Figure 3). The angle of the simulated branches changed, whereas other morphological parameters remained unchanged, and the TLA was 1.302 m^2^.

**Figure 4:**
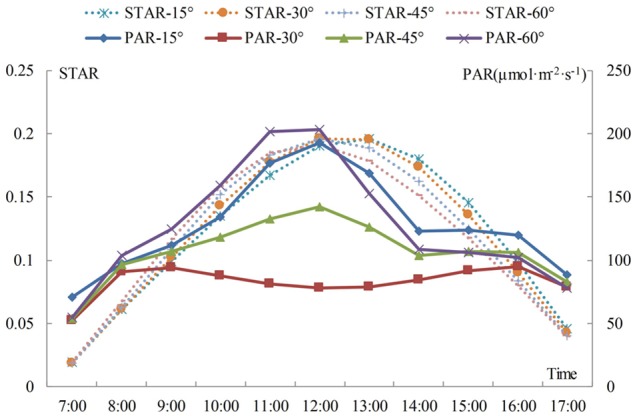
Daily variation in the STAR and total PAR for shoots in different angle between branch to trunk. The differently colored curves represent the variation in STAR and total PAR through time for different angles. The implementation represents the change of the PAR, and the dashed line represents the change of the STAR.

Figure 4 shows that the STARs and total PAR of branches with different angles tend to be similar when the change occurs during the day. The STAR first increases and then decreases from morning to afternoon. If the angle is 15°, the STAR reaches its highest point at 13:00, while the STAR of the other three angles reaches its highest point at 12:00. The results show that the efficiency of light interception is related to the relative angle of the branches and the solar azimuths. The STAR increases with an increase in branching angle before 11:00 and decreases with an increase in branching angle at a time ranging from 13:00 to 17:00. The change in the STAR is not obvious with an increase in the branching angle between 12:00 and 13:00; however, if the angle is 15° during this period, then the STAR presents an increasing trend, and the values for the other three angles present the opposite trend. The curve shows that the trend is the most stable if the angle is 30 to 45°. The average STAR for 1 day of the angle from 15 to 60° is: 0.122, 0.121, 0.119, 0.118. The average STAR increases when the angle decreases, and the average STAR is the largest when the angle is 15 or 30°, but the difference between the two angles is not obvious. The shoots may all grow upwards, and the angle of the level 2 scaffold branch may increase from 15 to 30°, with little change in the angle of the leaf.

The total PAR consists of solar direct radiation and sky diffuse radiation. The direct light intensity first increases and then decreases over time, the peak appears at approximately 12:00, and the diffuse light occurs bimodally at 8:00 and 17:00. This result is similar to that reported in a previous study (Tang et al., 2017). In the morning, PAR is mainly from diffuse light. By noon, the direct light increases sharply, with an increase in amplitude larger than the decrease in amplitude for diffuse light, which leads to an increase in the total radiation. At dusk, the difference between the decrease in direct radiation and the increase in diffuse radiation reduces the change in total PAR. Figure 4 shows that the total PAR is lower at the 30 and 45° angles. The maximum total PAR is obtained at an angle of 60° before 12:00 and at an angle of 15° after 12:00. The diurnal variation in STAR and PAR is characterized by an increase and then decrease, but higher PAR does not necessarily mean a larger STAR. The results show that the STAR is mainly affected by angle and slightly affected by the PAR intensity.

**Figure 5:**
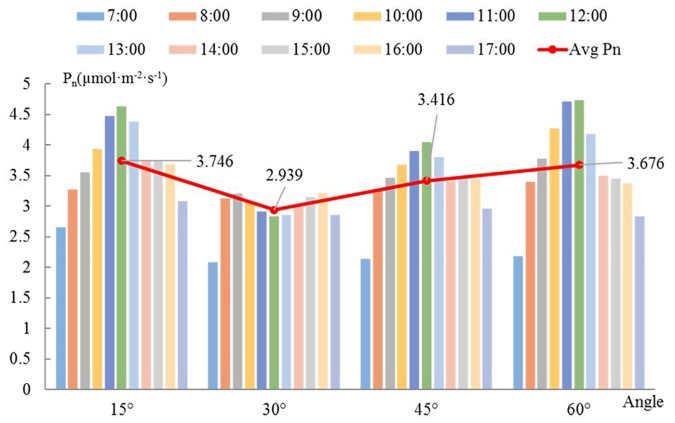
Diurnal change in net photosynthetic rate with angle. The bars with different colors represent different times. The red line shows the variation in the average net photosynthetic rate with the angle at a given hour of the day. The bars represent the daily average net photosynthetic rate of whole branches at different angles.

The diurnal variation of a single branch is similar among the different angles (Figure 5). The P_n_ increases over time in the morning; after 12:00, as the temperature increases, transpiration is enhanced and the stomas are closed, which causes the P_n_ to drop sharply. There is a relatively stable trend between 14:00 and 16:00, and the decrease in light energy after 16:00 causes the P_n_ to drop again. When the angle is 15 or 45°, the P_n_ reaches its daily maximum at 12:00. The P_n_ reaches a maximum at 11:00 when the angle is 60°. However, when the angle is 30°, P_n_ follows a bimodal distribution, whereas it follows a unimodal distribution for all other angles. Overall, the P_n_ is low when the angle is 30 or 45°. The maximum P_n_ occurs at the angle of 60° between 8:00 and 12:00, and the maximum P_n_ occurs at the angle of 15° at the other times. The red line in Figure 5 represents the trend of the average P_n_ per day with a change in angle. The average daily P_n_ of branches with an angle of 15° is the highest. There is no single linear relationship between the average P_n_ and the angle.

If the angle between the branch and the trunk is 15°, then the efficiency of light interception is the greatest, the variation over time is the lowest, and the PAR and P_n_ are the largest. Therefore, the scaffold branch angle should be kept small enough to increase the light interception of the loquat canopy; otherwise, the branches may be damaged, and the light interception cannot increase.

#### Shoots

**Figure 6:**
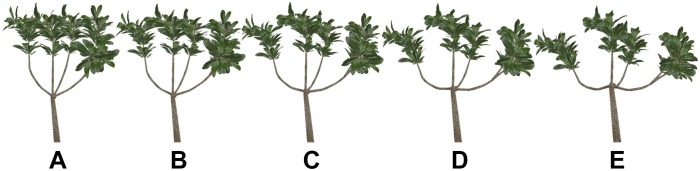
**(A–E)** The branch models with different angles between shoots. The figure shows the branches of leaves, showing the blade from different angles. The angle between branches gradually increase from AtoE.

With the abovementioned branches selected, the angle between the shoots rather than the angle between the branches and trunk was interactively adjusted, and other morphological parameters remained unchanged. The effects of five different topologies (different angles) on the distribution of light were analyzed by adding 10° to the angle between shoots each time (Figure 6). The angle between shoots ranges from 20 to 70°.

Similarly, the light interception was assessed in terms of the PAR, STAR, and P_n_ for five shoot forms. The results show that branching angle has a significant impact on PAR and P_n_, whereas the impact of branching angle on the STAR is small. With an increase in the angle, the direct PAR increases during the daytime from early morning to dusk, but the diffuse PAR decreases. From 8:00 to 12:00, the total PAR increases with an increase in the angle. At 7:00, 13:00 and 14:00, the total PAR increases with an increase in the angle, but when the angle increases to that shown in Figure 6E, the total PAR decreases. From 15:00 to 16:00, the total PAR increases across the angles shown in Figure 6A–C, and it decreases for the angles shown in Figure 6C–E. At 16:00, the total PAR increases slightly for the angles shown in Figure 6B,C. The average total PAR during the daytime increases with an increase in angle, and the average increase in total PAR for the angles shown in Figure 6D,E is lower than that for the other angles. The change in the STAR is similar over time, first increasing to the maximum value at 12:00 and then decreasing. At 7:00, for the angles shown in Figure 6A,B, the STAR decreases but not significantly; it increases with an increase in the angle between 8:00 and 10:00. At 11:00 and 12:00, the STAR increases for the angles shown in Figure 6A–C, and it decreases for those shown in Figure 6C–E. The STAR decreases with an increase in the angle between 13:00 and 17:00. The P_n_ gradually increases and then decreases over time. When the angle is one of those depicted in Figure 6A,B, the maximum P_n_ is reached at 12:00. For the other angles, the maximum P_n_ reaches its maximum at 11:00. The P_n_ increases gradually with an increase in the angle between 8:00 and 11:00, whereas the inverse occurs at 16:00. At 14:00, the P_n_ increases for the angles shown in Figure 6A–C and decreases for those shown in Figure 6C–E. At other times, the P_n_ increases for the angles shown in Figure 6A–D and decreases for those shown in Figure 6D,E.

Between the successive pairs of angles shown in Figure 6A–E, the average PAR increases by 5.041, 7.97, 5.291, and 0.926 μmol m^-2^ s^-1^, respectively; the average STAR decreases slightly. The average P_n_ increases for the angles shown in Figure 6A–D and decreases for those shown in Figure 6D,E, but the decrease is not significant. The average PAR and P_n_ increase with an increase in the angle, but the average STAR remains stable. For the angles shown in Figure 6B,C, the growth rate of average PAR and average P_n_ is the highest. As the angle continues to increase, the amount by which these values increase does not become significantly larger.

### At the Canopy Scale

#### Branch and Trunk

**Figure 7:**
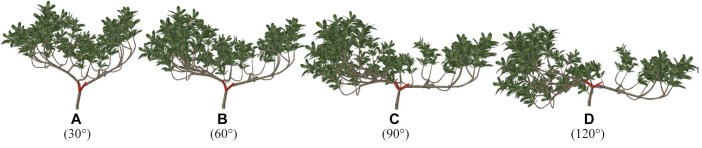
**(A–D)** Virtual representation of loquat morphology for different angles. The angle of the red line represents the angle below, that is, the angle of the level 2 scaffold branch in the three-dimensional space. The model was generated by the fast shaping and pruning function. The model information is the same for all scenarios, except for the angle.

Through plastic pruning and branch bending achieved by adjusting the branch angle, which are methods used to adjust branch morphology in the Supplementary Figure S1, four 3D architectural canopy models of loquat were generated. Figure 7 shows loquat models with different angles between two branches, and their leaf areas are all 8.093 m^2^. The light distribution was simulated within the canopies, and light interception was evaluated in terms of STAR, PAR, and P_n_ to explore the most suitable range of angles.

**Figure 8:**
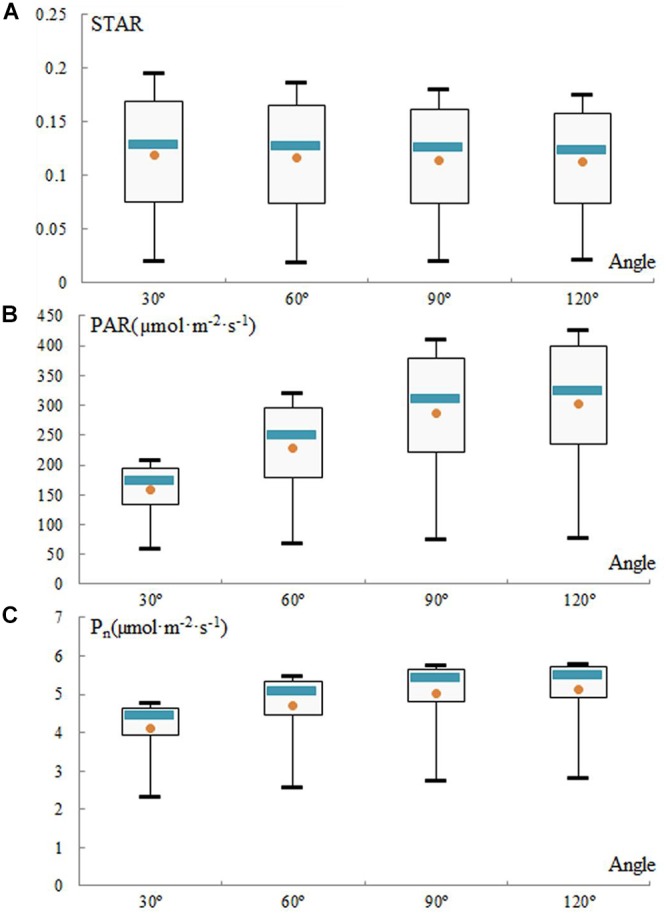
Evolution of silhouette to total area ratio (STAR) **(A)**, photosynthetically active radiation (PAR) **(B)**, and net photosynthetic rate (Pn) **(C)** for simulated trees from 7:00 to 17:00 daily in the experiment employing different geometry and morphology, when varying the angle parameters. Each box extends from the lower to upper quartile values, with a blue line at the median and a dot at the mean, respectively.

In these canopy models, the STAR, PAR, and P_n_ exhibited opposite diurnal patterns. The distribution of the STAR of the simulated canopies is represented every hour from 7:00 to 17:00 (Figure 8A). The mean STARs range from 0.119 at 30° to 0.112 at 120° and were consistent among the angles. The STAR decreases with an increase in the angle, and it does not change obviously between 60 and 90°.

In contrast, for angles from 30 to 120°, the mean PAR and mean P_n_ increase from 157.52 to 303.42 μmol m^-2^ s^-1^ and from 4.13 to 5.11 μmol m^-2^ s^-1^, respectively (Figure 8B,C). However, these increases, which were very rapid from 30 to 60°, were attenuated between 90 and 120°. The change in PAR was consistent with the change in P_n_. The total PAR gradually peaked between 12:00 and 13:00 and then decreased. The higher the angle is at any time, the higher the PAR is due to less leaf shading. As consequence, the greater the angle is, the higher the values of PAR and P_n_ are, but the increase in the angle from 60 to 120° is not obvious.

In other words, the angle has a significant impact on the optimum light interception efficiency. Increasing the angle between shoots will increase the values of STAR, PAR, and P_n_ within limits. The topological relationship between shoots of loquat is satisfactory in Figure 8B,C. Therefore, high-light-efficiency canopy architecture is preferred, and 60 and 90° thus represent the ideotypes of loquat (Figure 8B,C). The relative canopy height also decreases when the angle increases, and the ideal plant types have an angle of 60 or 90°, which also indirectly indicates that suitable dwarfing of the loquat canopy is beneficial for improving the efficiency of light interception of loquat.

#### Changes in Other Parameters

**Figure 9:**
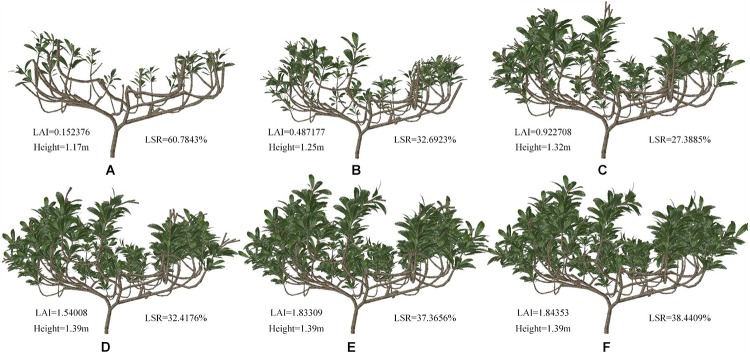
**(A–F)** Schematic representation of loquat morphology for different pruning schemes. The LAI, height and ratio of number of long shoots to number of total shoots (LSR) data are shown in the picture.

The complete tree model was constructed with different geometries and topologies through shaping and pruning. Figure 9 shows samples of these simulated tree architectures. The top shoot diameter is expected to have an impact on branch bending and leaf orientation. The leaf density is determined by both the intervals between leaves, as determined by internode length, and the branching behavior of the canopy. Consequently, we chose four geometrical traits related to these aspects, namely, height, long shoot ratio (LSR), TLA and leaf area index (LAI), to investigate the complex influences of these contributors to leaf density on the whole-tree STAR, PAR, and P_n_. The branches were divided two types: short (fewer than 10 nodes) and long (more than 10 nodes).

Figure 9A–F can be regarded as the tree shapes for different pruning schemes. The morphological structure of trees shown in Figure 9A–C mainly increases the number of shoots and leaves. Figure 9D–F show elongated branches with little change in leaf area. We analyzed these geometric traits (Figure 9 and Table 3). Table 3 shows some parameters of tree morphology depicted in Figure 9. As shown in Table 3, regardless of the underlying assumption regarding leaf, shoot and branch position within the trees, the mean STARs estimated from simulated tree molels decrease with the number of branches and leaves, with stable variability. These results are in agreement with those observed by Han et al. (2012) and Silva et al. (2014a). The mean values estimated at the whole-tree level ranged from 0.417 to 0.210 and tended to stabilize among D, E, and F, where they ranged from 0.125 to 0.120. This pattern resulted from the low overlap between leaves, as shown in Figure 9A,B, and the relatively high overlap shown in Figure 9D–F. In contrast, the height of the simulated tree increased from 1.17 to 1.39 m, and the TLA ranged from 0.610 to 10.792 m^2^. With an increase in TLA, the LAI and average P_n_ gradually increased, indicating that leaf area is an important factor affecting P_n_. However, the constant increase in TLA compared to the relatively stable STARs suggests that increased STAR does not result only from the increase in leaf area. The average PAR does not change consistently with leaf area. The decrease in average PAR for the morphological structure depicted in Figure 9D indicates that PAR is related not only to leaf area but also to the proportion of long shoots. An appropriate reduction in the number of long branches is beneficial for the acquisition of PAR.

**Table 3:** Comparison of morphological and light distribution parameters.

Different morphology	LSR (%)	TLA (m^2^)	LAI	Avg STAR	Avg PAR (μmol ⋅ m^-2^ ⋅ s^-1^)	Avg P_n_ (μmol ⋅ m^-2^ ⋅ s^-1^)
A	60.78	0.610	0.152	0.417	185.04	4.421
B	32.69	2.157	0.487	0.210	221.32	4.684
C	27.39	4.787	0.923	0.159	237.09	4.790
D	32.42	8.468	1.540	0.125	211.97	4.605
E	37.37	10.181	1.833	0.121	231.50	4.746
F	38.44	10.792	1.844	0.120	240.55	4.802

The correlations between light interception and the geometric parameters were analyzed. The LSR was positively correlated with the STAR, and the correlation coefficient was 0.849. The LSR was negatively correlated with PAR and P_n_, and the correlation coefficients were 0.414 and 0.785, respectively. The results showed that the LSR had little effect on PAR but had a strong influence on the STAR and P_n_. Tree height, TLA and LAI were negatively correlated with the STAR, and tree height had the most important effect. Thus, the taller the tree is, the lower the efficiency of light interception is, which was consistent with the conclusion of previous studies that loquat trees should be dwarfed to improve ventilation and light transmission (Qiu et al., 2006; Pan, 2017). The correlation of tree height, TLA and LAI with PAR was as high as 0.949, 0.997, and 0.994, respectively. These results showed that tree height and leaf area had significant effects on PAR, and leaf area played a major role. The correlations of tree height, TLA and LAI with P_n_ were 0.702, 0.638, and 0.624, respectively. These results indicate that tree height and leaf area have a relatively important influence on thephotosynthetic rate.

## Discussion

Virtual plants would be a novel approach for quantitatively analyzing the light interception of the canopy, thereby aiding in the design of tree architecture. Based on our previous studies (Tang et al., 2011, 2017, 2018), the software LSTree has been extended for tree-type design to examine the light interception of a canopy by integrating a radiosity model and modified rectangular hyperbola model. The interactive design module was implemented using the pseudo color offscreen rendering method; this method can obtain objects in large graphics quickly, which contributes to the interactive editing of 3D tree models. The modeling process requires the user to have some knowledge of computers and botany, i.e., understanding and defining L-system rules, which is a usage restriction. According to the tree morphological characteristics derived from field measurements or the literature, a new 3D tree model can be generated by defining L-system-produced rules using LSTree. The 3D tree model is faithful to botanical principles and is similar to the actual morphological structure of the tree. Moreover, the 3D model can be pruned, and its angles (between shoots, between branches or between branches and the trunk) can be adjusted to generate various canopy type models. Based on existing canopy information (available cultivars in an orchard in Yunxiao County, Fujian Province), various loquat models were constructed through shaping and pruning. The models are adaptations of existing cultivars rather than complete redesigns. The pruning strategy is accepted by end-users (Andrivon et al., 2013). This method provides an effective aided-analysis technology for training (e.g., branch drawing) in the field to reduce the damage to plants caused by incorrect operations.

Ideotypes of plants are ideal combinations of traits in specific genotypes used to achieve predefined production goals, such as high yield, high light efficiency, and low disease incidence. An ideotype can only be defined relative to a goal and/or one set of constraints (Andrivon et al., 2013). The main constraints are the genetic or plastic variability available within the species considered. In the present study, high light efficiency is one objective of optimum design. The STAR, PAR, and the P_n_ were considered major performance metrics for evaluating the light interception of the canopy. Reasonable pruning is conducive to producing more efficient and homogeneous light absorption in the inner canopy. Compared with the natural (unpruned) canopy, the pruned canopy has significantly greater light interception at the level of the whole tree canopy. However, the effects vary depending on the branch angle. The models of loquat at different scales were analyzed from many perspectives. The results showed that if the single-branch angle between the level 2 scaffold branch and trunk is approximately 15°, a better light distribution can be obtained. The angle among shoots should be sufficiently large to ensure that there are enough gaps between the leaves to allow light to pass through, but an angle that is too large will either increase the light distribution or damage the morphology and growth. Angles among the level 2 scaffold branches ranging from 60 to 90° might be more appropriate for loquat. In addition, proper pruning should be carried out during the growth process to ensure that the efficiency of light interception and photosynthesis is maintained at an appropriate level. These findings reveal that branch angle and the number of branches are the major factors affecting light distribution and lay a foundation for further studies of photosynthetic products in different light environments and for different tree morphologies. In conclusion, high light interception is controlled by the angle of the branches.

The selection of ideal architectural types provides a mode and direction for further creative research on 3D visual structures and thus provides theoretical and technical support for tree architectural design. This study only simulated light interception and photosynthesis in 3D canopy models, for which some of the correlations were clear, and significant differences were identified between different types of architectures. Due to technical limitations, it is impossible to precisely measure the light interception of foliage within a canopy in the field. Therefore, despite the lack of light interception measurement for validation, the method and corresponding software LSTree can facilitate precise analysis of light interception in virtual canopies. Here, we analyzed light interception at the scale of a single branch and a single tree. Further research should be carried out on light interception at the orchard level in the future.

## Data Availability

The raw data supporting the conclusions of this manuscript will be made available by the authors, without undue reservation, to any qualified researcher.

## Author Contributions

DY designed the experiments, developed the program, constructed the 3D model, analyzed the data, and wrote the first draft of the manuscript. LT conceived and designed the study and revised the manuscript. CC conceived the study. All authors discussed the results and revised the manuscript.

## Conflict of Interest Statement

The authors declare that the research was conducted in the absence of any commercial or financial relationships that could be construed as a potential conflict of interest.

## Supplementary Material

FIGURE S1 Field adjustment of loquat morphology in the Yunxiao County in Fujian Province, China, on January 18–22, 2016. The picture shows branch drawing of operation on loquat tree. The bags in the picture are bagging the fruit.
