# Supplementary figures and images for: Optimal Design of Plant Canopy Based on Light Interception: A Case Study With Loquat
Source: Front Plant Sci. 2019 Mar 26;10:364. doi: 10.3389/fpls.2019.00364 (PMC6443822; doi:10.3389/fpls.2019.00364)

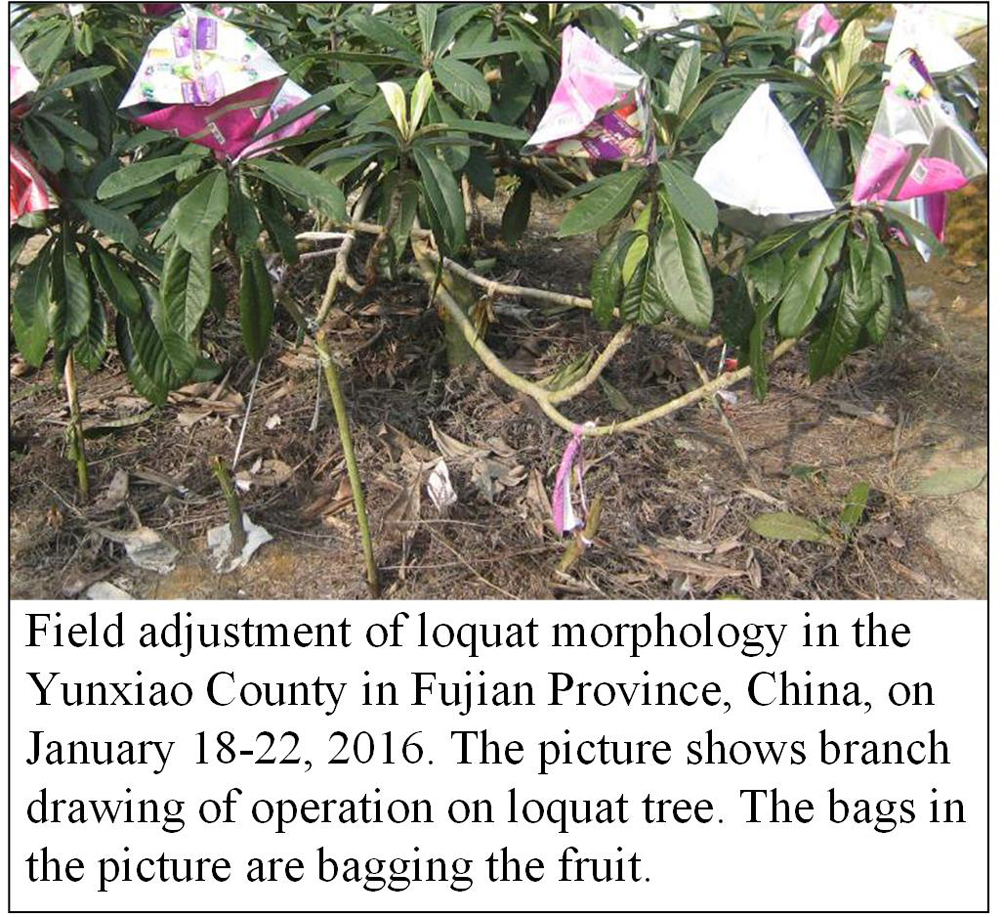

Supplement: FIGURE S1 — Field adjustment of loquat morphology in the Yunxiao County in Fujian Province, China, on January 18–22, 2016. The picture shows branch drawing of operation on loquat tree. The bags in the picture are bagging the fruit. [file Image_1.JPEG]
